# Early prediction of the bactericidal and bacteriostatic effect of imipenem and doxycycline using tabletop scanning electron microscopy

**DOI:** 10.3389/fcimb.2024.1431141

**Published:** 2024-08-29

**Authors:** Omar Zmerli, Alma Hodzic, Sara Bellali, Eid Azar, Jacques Bou Khalil

**Affiliations:** ^1^ IHU – Méditerranée Infection, Marseille, France; ^2^ Aix Marseille Univ, MEPHI, Marseille, France; ^3^ Division of Infectious Diseases, Saint George Hospital University Medical Center, Beirut, Lebanon

**Keywords:** scanning electron microscopy, tabletop SEM, bactericidal effect, bacteriostatic effect, rapid methods in microbiology, bacterial morphology

## Abstract

**Introduction:**

Our work aims at establishing a proof-of-concept for a method that allows the early prediction of the bactericidal and bacteriostatic effects of antibiotics on bacteria using scanning electron microscopy (SEM) as compared to traditional culture-based methods.

**Methods:**

We tested these effects using Imipenem (bactericidal) and Doxycycline (bacteriostatic) with several strains of sensitive and resistant *Escherichia coli*. We developed a SEM-based predictive score based on three main criteria: Bacterial Density, Morphology/Ultrastructure, and Viability. We determined the results for each of these criteria using SEM micrographs taken with the TM4000Plus II-Tabletop-SEM (Hitachi, Japan) following an optimized, rapid, and automated acquisition and analysis protocol. We compared our method with the traditional culture colony counting gold standard method and classic definitions of the two effects.

**Results:**

Our method revealed total agreement with the CFU method and classic definition by visualizing the effect of the antibiotic at 60 minutes and 120 minutes using SEM.

**Discussion:**

This early prediction allows a rapid and early identification of the bactericidal and bacteriostatic effects as compared to culture that would take a minimum of 18 hours. This has several future applications in the development of SEM-automated assays coupled to machine learning models that identify the antibiotic effect and facilitate determination of bacterial susceptibility.

## 1 Introduction

The discovery of antibiotics revolutionized modern medicine, and despite the previous delineation of their mechanisms of actions, some interactions with bacteria remain challenging to observe and explain (Brown and Wright, 2016). Several approaches are used to classify and differentiate antibiotics in an attempt to improve their use. From a microbiological point of view, one common classification relies on their ability to alter bacterial metabolism (Stokes et al., 2019), either by inhibiting their growth (bacteriostatic), or by inducing their death (bactericidal) (Brown and Wright, 2016). The exact definition of these two basic microbiological principles remains elusive due to a wide range of factors related to bacterial diversity, pharmacological properties of antibiotics, and deficient detection methods (Stokes et al., 2019; Orrell-Trigg et al., 2024). Moreover, there is evidence that these effects are not dichotomous, as certain molecules have exhibited both bactericidal and bacteriostatic behaviors in different settings, hosts, and at different concentrations (Pankey and Sabath, 2004; Verma et al., 2022).

Nevertheless, the bactericidal/bacteriostatic classification remains, to this day, the cornerstone for developing methodologies to perform bacterial susceptibility testing (Reller et al., 2009) and identify the mechanism of action of antibiotics from the moment of their discovery until after they are administered to the patients (Peterson and Shanholtzer, 1992). The ability to rapidly predict antibiotic effects holds important clinical and research significance. This can potentially improve patient outcomes by accelerating access to effective treatment and promoting the judicious selection of antibiotics. Many methods have been developed to discover the mechanism of action of antibiotics and recognize bacterial viability status, including viability stains/dyes, molecular biology methods, and microfluidic assays (Baranova et al., 2023). These techniques often require extended turnaround times and may lack sensitivity or specificity. More sophisticated techniques exist for research purposes, but these require higher costs, specialized expertise, and involve intricate data analysis approaches (Schäfer and Wenzel, 2020).

Currently, multiple variations of the classic broth microdilution assays are used in microbiology laboratories for identifying bactericidal versus bacteriostatic effects. In general, the bacteriostatic effect is commonly revealed by measuring the Minimum Inhibitory Concentration (MIC), which is the lowest concentration of antibiotic that prevents visible growth of bacteria, essentially inhibiting bacterial multiplication (Peterson and Shanholtzer, 1992; Reller et al., 2009). On the other hand, the bactericidal effect is universally determined by measuring the Minimum Bactericidal Concentration (MBC), which is the lowest concentration of antibiotic that reduces more than 99.9% of viable bacteria (3-log_10_ reduction) as compared to the initial bacterial concentration at 18 hours (Peterson and Shanholtzer, 1992). Despite their supposed precision, these definitions remain controversial and difficult to standardize due to variations in culture media, inoculum size, and incubation times (Peterson and Shanholtzer, 1992; Institute C and LS, 1999; Pankey and Sabath, 2004).

Significantly, recent advancements in electron microscopy, particularly the development of compact and user-friendly tabletop scanning electron microscopes (SEMs), have revolutionized the study of bacterial morphology and viability (Haddad et al., 2021; Zmerli et al., 2023). These new generation SEMs enable the direct observation of a broad range of samples with minimal preparation steps, compared to the more complex and time-consuming protocols required by older electron microscopy techniques.

Therefore, we revisit these definitions by introducing a more stable and rapid method for direct observation of antibiotic interaction with bacteria using tabletop scanning electron microscopy (SEM). Consequently, we leverage the real-time visualization capabilities of SEM to establish the proof-of-concept of a rapid and simplified prediction of bactericidal and bacteriostatic effects at earlier time-points compared to traditional culture methods for two major antibiotics.

## 2 Materials and methods

### 2.1 Antibiotic and media preparation

We used freshly prepared aqueous 1mg/mL solutions of imipenem and doxycycline hyclate. (Sigma-Aldrich) Mueller-Hinton Broth II (MHB) (Millipore, Sigma-Aldrich) was prepared according to manufacturer instructions (KGaA M), and filtered at 0.22µm.

### 2.2 Bacterial strain selection

We selected six strains of *Escherichia coli* based on their susceptibility to imipenem and doxycycline, as detailed in **Table 1**. Strains were ordered from the Collection de Souches de l’Unité des Rickettsies (CSUR, WDCM 875) and the American Type Culture Collection (ATCC). All chosen strains originated from clinical samples. We verified the identity of the selected strains using MALDI-TOF MS (Seng et al., 2009) (matrix-assisted laser desorption/ionization time-of-flight mass spectrometry; Bruker Daltonics, Germany), and determined strain susceptibility and exact MIC using the E-test (bioMérieux, France) (Etest Application Guide). We performed our experiment in duplicates for each of the chosen species.

**Table 1:** Selected bacterial strains for predicting bactericidal and bacteriostatic effects using SEM with MIC levels for imipenem & doxycycline.

	Imipenem MIC (Profile)	Doxycycline MIC (Profile)
***Escherichia coli* Q5585**	**0.125 mg/L (Sensitive)**	**1.5 mg/L (Sensitive)**
***Escherichia coli* ATCC25922**	**0.125 mg/L (Sensitive)**	**3 mg/L (Sensitive)**
***Escherichia coli* Q2155**	0.5 mg/L (Sensitive)	**96 mg/L (Resistant)**
***Escherichia coli* Q0385**	0.125 mg/L (Sensitive)	**>256 mg/L (Resistant)**
***Escherichia coli* Q9367**	**3 mg/L (Resistant)**	12 mg/L (Resistant)
***Escherichia coli* Q9382**	**2 mg/L (Resistant)**	12 mg/L (Resistant)

Strain-Antibiotic combination tested in bold.

### 2.3 Experimental protocol

**Figure 1:**
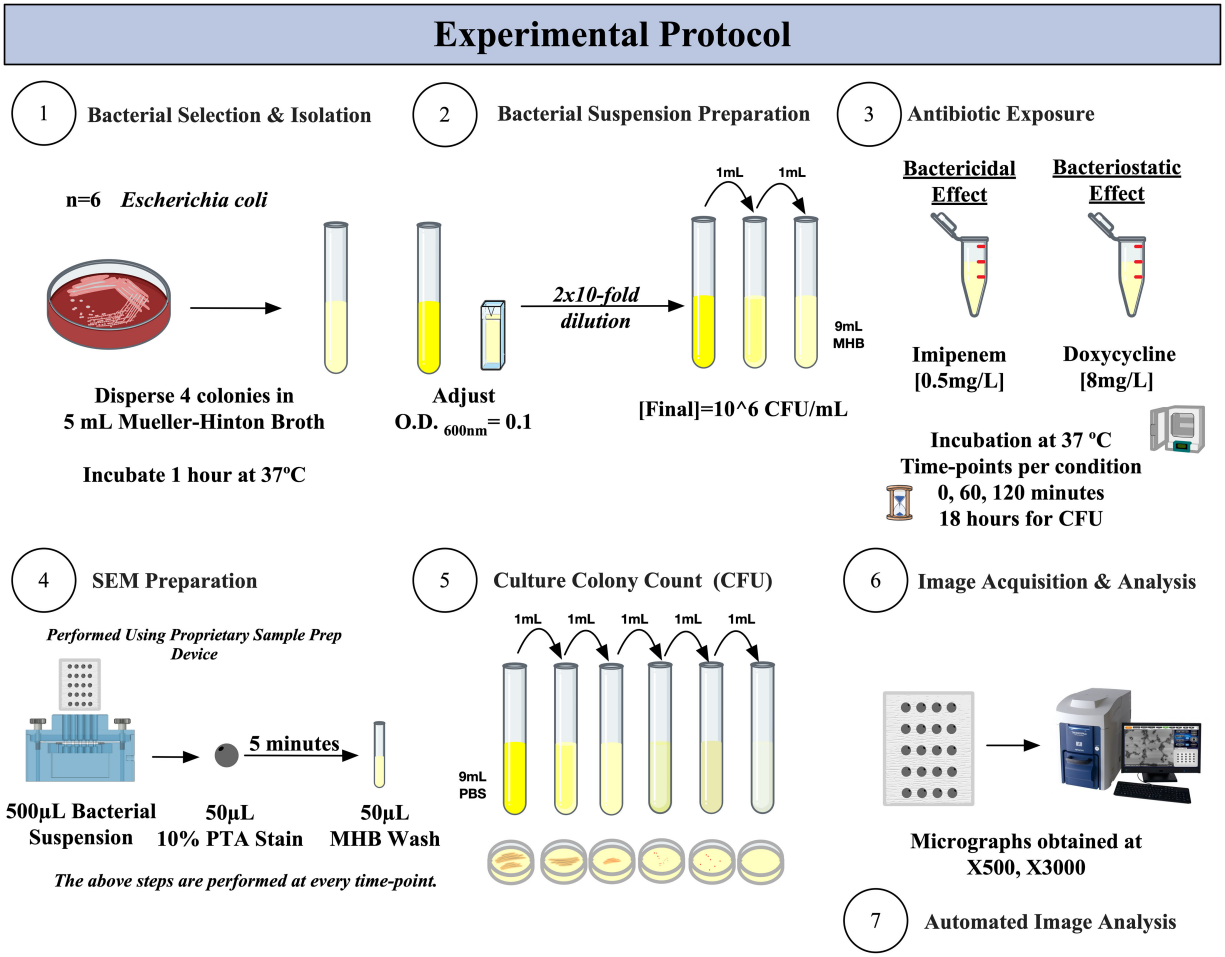
Experimental protocol for SEM-PTA and culture colony counting (CFU) for the determination of bactericidal and bacteriostatic effects of imipenem and doxycycline.

**Figure 1** summarizes the experimental protocol we designed to demonstrate the bactericidal and bacteriostatic effects using SEM, as compared to the traditional culture colony counting method.

#### 2.3.1 Antibiotics exposure protocol

We cultured the chosen isolates on Columbia agar + 5% sheep blood (bioMérieux, France) overnight; then resuspended four colonies in 4mL of MHB and incubated for 1 hour at 37°C. The bacterial suspension was then adjusted to an optical density (O.D._600_) of 0.10 using the Ultrospec 10 cell density meter (Biochrom, UK). The suspension was then diluted 20-fold to obtain a bacterial concentration corresponding to ~10^6^ CFU/mL.

For testing the bactericidal effect, 5mL of bacterial suspension was mixed with 2.5µL of imipenem [cut-off [0.5mg/L] (Testing EC on AS)]. As for testing the bacteriostatic effect, 5mL bacterial suspension was mixed with 40µL of doxycycline [cut-off [8mg/L] (Testing EC on AS)]. These were then incubated at 37°C with shaking at 150 rpm, with progressive sampling of the tubes at the following times: 0, 1, 2, and 18 hours – reflecting early time-points suitable for developing a rapid detection versus the gold standard reading which is normally performed at a minimum of 18 hours of incubation (ISO, 2021).

#### 2.3.2 Bacterial viability determination

At every time-point, we applied two bacterial viability assays, as follows:

##### 2.3.2.1 Bacterial viability by culture colony count

Visible colonies were counted, and the CFU/mL concentration was calculated as previously described. Briefly, serial decimal dilutions were prepared under aerobic conditions with Phosphate Buffered Saline (PBS) (Life Technologies, Paisley, United Kingdom), and each condition was plated in duplicate onto Columbia blood agar plates under a sterile cabinet. The agar plates were incubated for 24 hours at 37°C. Culturable colony counts were used to determine viable bacterial concentration at every time-point per condition.

##### 2.3.2.2 Bacterial viability by SEM-PTA assay

A polycarbonate hydrophilic iso-pore track-etched membrane filter (pore diameter 0.2 µm) (ipPORE, it4ip) was used as a cell support. The membrane was prepared as previously described (Hisada et al., 2023) At every time-point, 500µL of bacterial suspension were stained with 50µL of aqueous 10% Phosphotungstic Acid (PTA) (Sigma-Aldrich, St. Louis, MO, USA) at pH 7 for 5 minutes, followed by a single 50µL wash with MHB. This staining allows the discrimination of live and dead bacteria based on their contrast (electron density on SEM imaging), as previously described (Hisada et al., 2023; Zmerli et al., 2023).

### 2.4 Electron micrograph acquisition

We used the TM4000Plus II Tabletop scanning electron microscope (SEM) (Hitachi High-Tech, Japan) to obtain micrographs, with the following acquisition settings: 5kV accelerating voltage and BSE detector. We acquired the micrographs at low magnification (x500) for bacterial density analysis and high magnification (x3000) for morphologic and ultrastructure analysis, using identical settings per condition. Images were acquired from randomly selected regions of the sample to enhance representation and homogeneity of detection. Acquisition settings are visible on each micrograph in the following format: Instrument, Accelerating Voltage, Working Distance, Magnification, and Detector. We performed our experiment in duplicates for each of the chosen species.

### 2.5 Post-acquisition analysis & SEM-based predictive scoring

**Figure 2:**
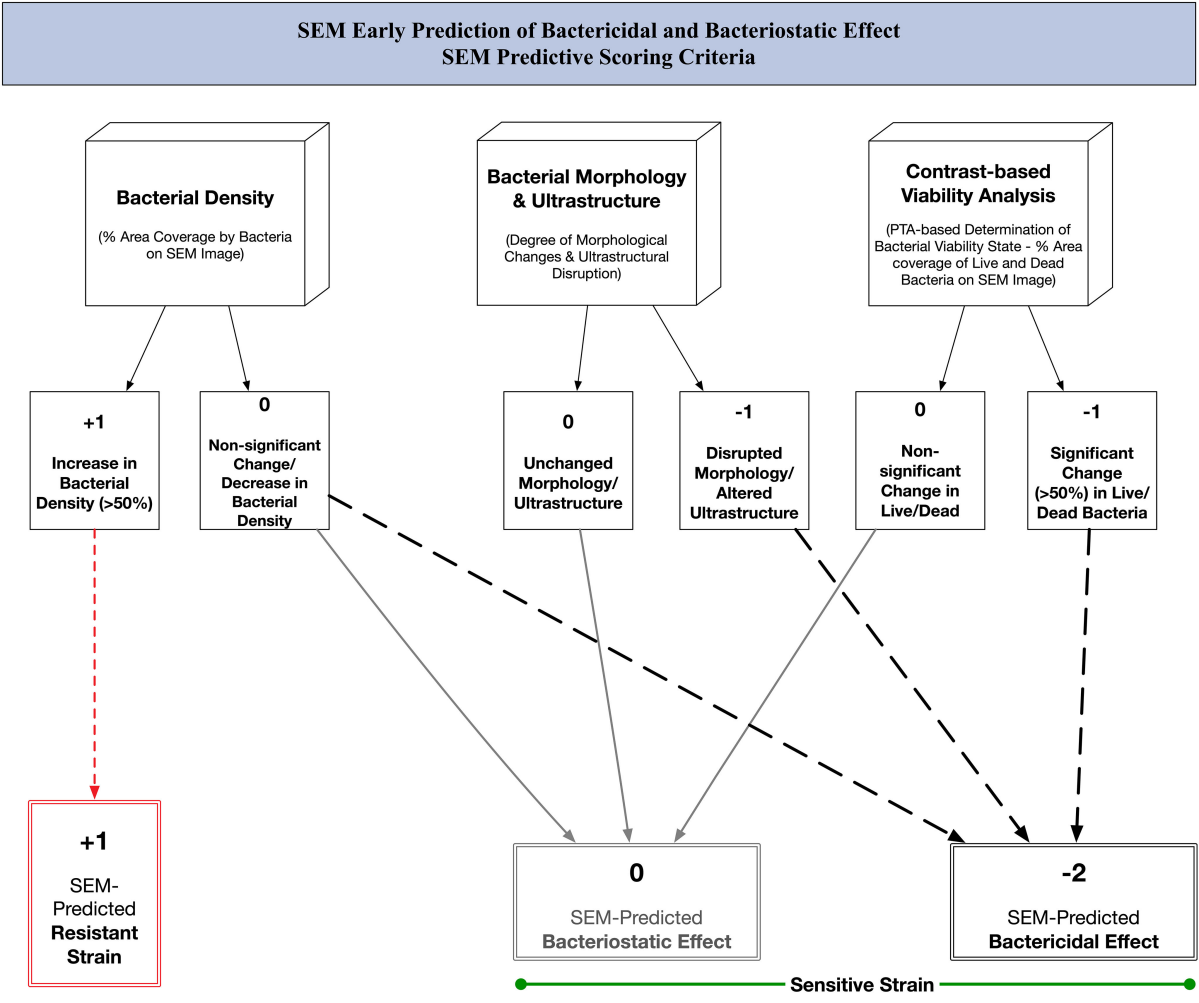
SEM predictive scoring criteria: algorithm for early prediction of bactericidal and bacteriostatic effect of antibiotics using scanning electron microscopy.

We built our post-acquisition analysis protocol taking into consideration all available variables from our images. Our protocol incorporated three major criteria, each yielding a percentage result that can be compared to trends seen with traditional culture-based methods. We integrated the outcomes of this three-criteria analysis to develop a SEM-based predictive score. This score enabled us to forecast the bactericidal or bacteriostatic effects of Imipenem and Doxycycline, respectively, on sensitive strains and to observe resistant strains. (**Figure 2**) We defined a significant change as being more than 50% different from the baseline. We performed our analysis using Image-Pro 10.0.15. (Media Cybernetics) by means of the automated smart image segmentation and 2D-object quantification tools. (**Supplementary Data 1**) The analysis was applied to low magnification images (x500) and high magnification images (x3000) for each condition at every time-point.

#### 2.5.1 Bacterial density analysis

We define percent bacterial density (%BD) as the percentage area occupied by bacteria on each SEM image.

#### 2.5.2 Bacterial morphology and ultrastructure analysis

PTA staining improved our ability to visually track ultrastructure changes following antibiotic exposure and, most importantly, define morphologic changes in bacterial cells, not limited to cell inflation, fusion, deformation, and lysis.

#### 2.5.3 Contrast-based viability analysis

Bacterial viability detection using the SEM-PTA assay was done by differentiating bacterial cells based on PTA-staining: Live bacteria appear dark/electron-lucent, while dead bacteria appear bright/electron-dense; as previously described (Hisada et al., 2023; Zmerli et al., 2023). This difference in contrast was defined using the Smart Segmentation tool, allowing the identification of percent live (%Live) and percent dead (%Dead) bacteria per SEM image.

### 2.6 Statistical analysis

We calculated the means for %BD, %Live, %Dead, and CFU/mL results among all tested isolates, in duplicate, per susceptibility profile. We graphed the means and standard deviations using Prism 9.5.0. Further statistical analysis was not done because the results were self-evident. Combining the scores corresponding to each criterion enabled the calculation of a final score and the prediction of the antibiotic’s effect.

## 3 Results

### 3.1 Bactericidal effect – exposure to imipenem

**Figure 3:**
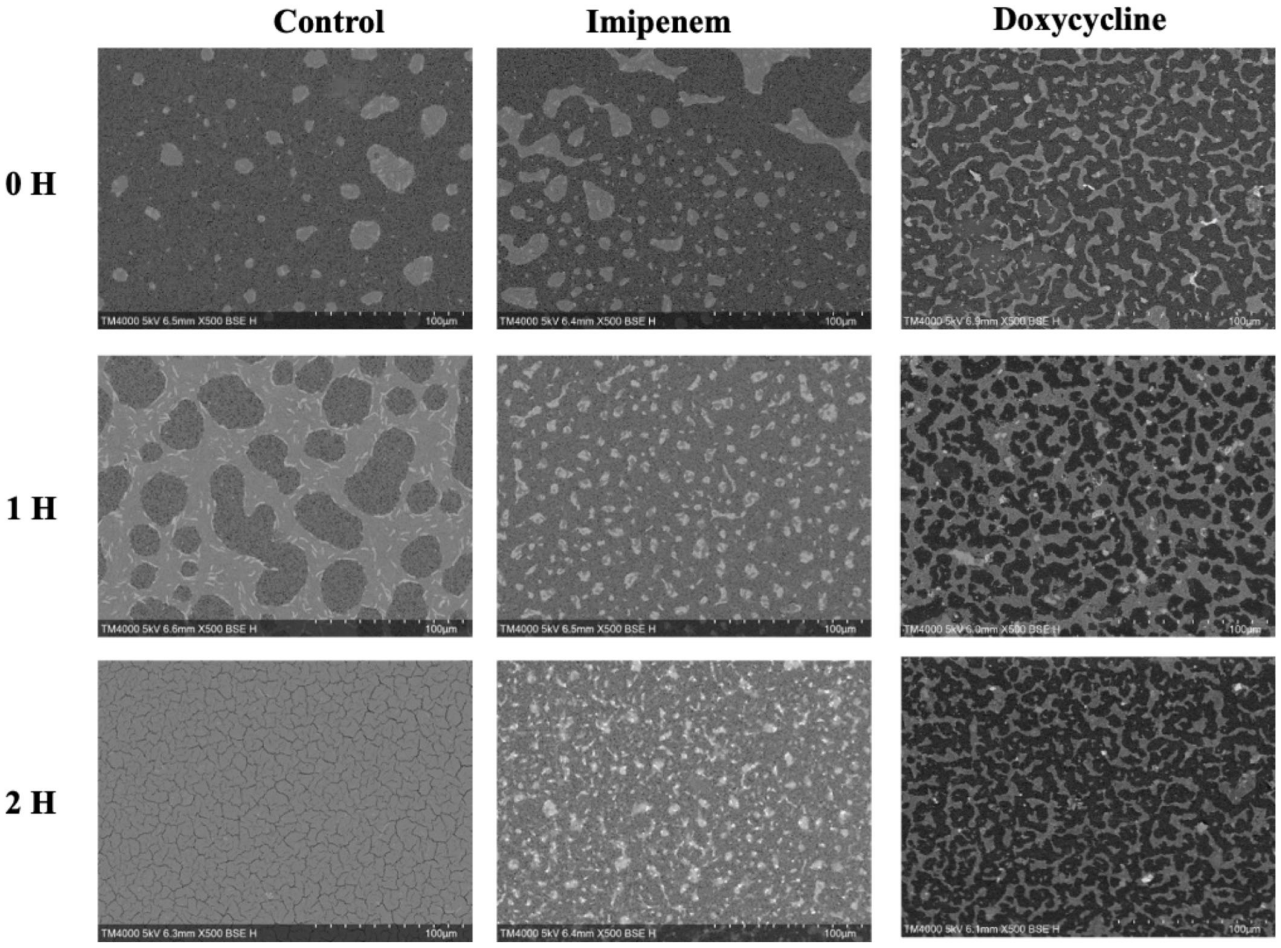
Growth density analysis between Bactericidal and Bacteriostatic Effect on Sensitive *E. coli* strains on SEM micrographs taken at Low Magnification at Early Time-points. Acquisition settings and magnification are noted on micrographs.

**Figure 4:**
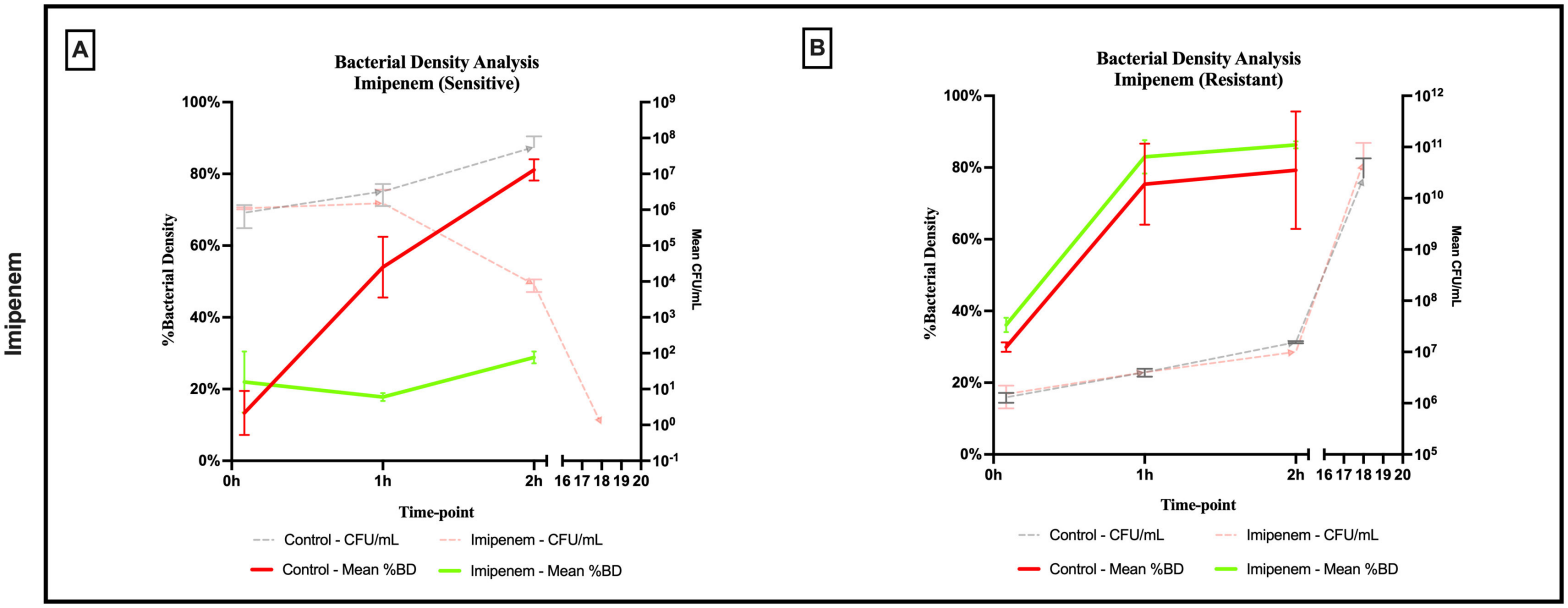
Percent bacterial density means analysis of bactericidal effect of imipenem on **(A)** sensitive and **(B)** resistant strains of *E.coli* using scanning electron microscopy compared to culture colony counts (CFU). Error bars represent the standard deviation (SD).

**Figure 5:**
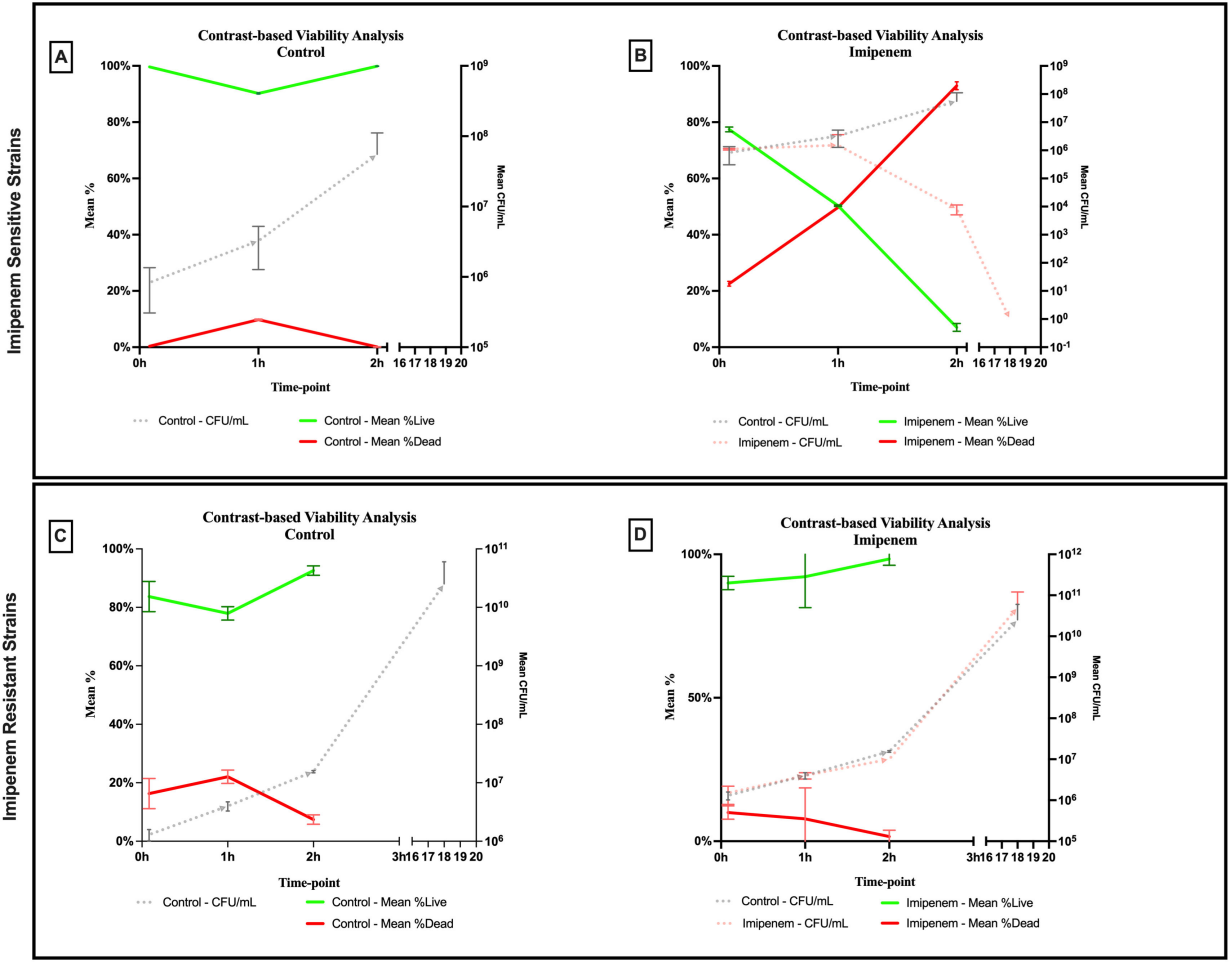
Contrast-based viability analysis of bactericidal effect of imipenem on **(A, B)** sensitive and **(C, D)** resistant strains of *E.coli* using scanning electron microscopy compared to culture colony counts (CFU). Error bars represent the standard deviation (SD).

**Figure 6:**
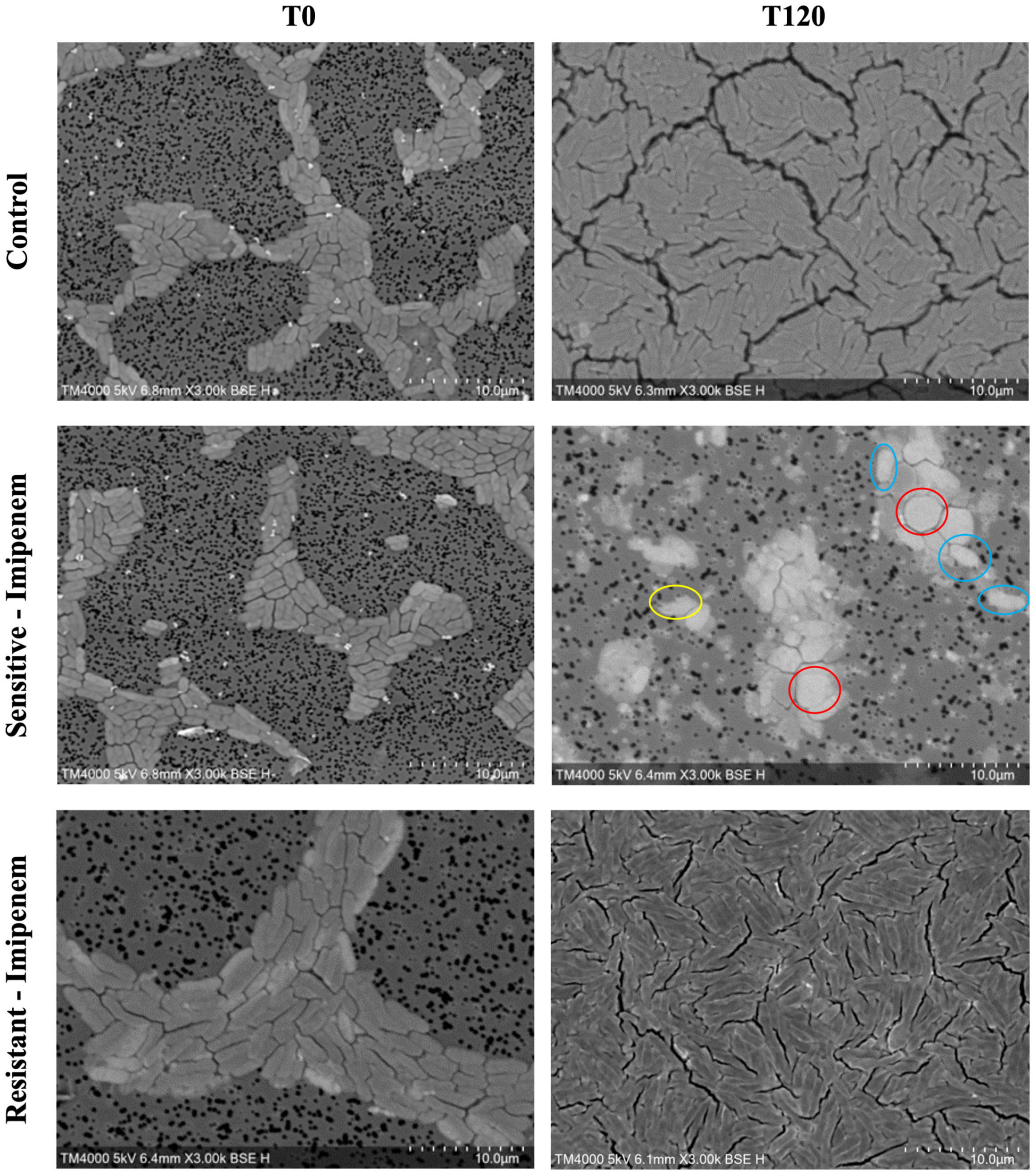
Morphologic/ultrastructural changes demonstrating the bactericidal effect of imipenem on sensitive and resistant E.coli strains using scanning electron microscopy.

A significant difference was observed across all axes of our analyses for the Imipenem sensitive strains of *E. coli*. Bacterial density analysis revealed a striking decrease in mean %BD two hours following Imipenem exposure (22.92% ± 1.57) as compared to the control (82.23% ± 0.06). (**Figures 3**, **4A**) Contrast-based viability analysis also revealed a significant rise in the mean %Dead bacteria (92.94% ± 1.39) in the Imipenem exposed bacteria, contrary to the near disappearance of dead bacteria in the control (0.1% ± 0.04). (**Figures 5A, B**) Similarly, following exposure to Imipenem, morphological changes became evident as early as 60 minutes. At 120 minutes, we could clearly identify deformed bacteria (red circle), in addition to inflated (blue circle) and lysed (yellow circle) bacterial cells. (**Figure 6**) A parallel trend was observed in the culture colony counting method, revealing a 99% growth reduction at two hours, and a 100% growth reduction at 18 hours in the Imipenem exposed bacteria, in contrast to a substantial >3-log10 growth in the control at 18 hours. (**Figures 4**, **5**) Applying the SEM Predictive Score based on the combined results of the respective criteria provides a score of -2 (Bactericidal Effect) for all tested sensitive strains.

Among the Imipenem resistant *E. coli* strains, we observed a parallel trend between the Imipenem exposed bacteria and the controls. The resistant bacteria maintained their original morphology following exposure to Imipenem and with no significant structural or morphological changes (**Figure 6**). Similarly, the culture colony count at 120 minutes showed a parallel growth between the control and the Imipenem exposed bacteria; further confirmed by the relatively stable percentage of dead (electron-dense) cells, with a complete predominance of living bacteria visible at 120 minutes (**Figures 4B**, **5C, D**). Applying the SEM Predictive Score based on the combined results of the respective criteria provides a score of +1 (Resistant Strain) for all tested resistant strains.

### 3.2 Bacteriostatic effect – exposure to doxycycline

**Figure 7:**
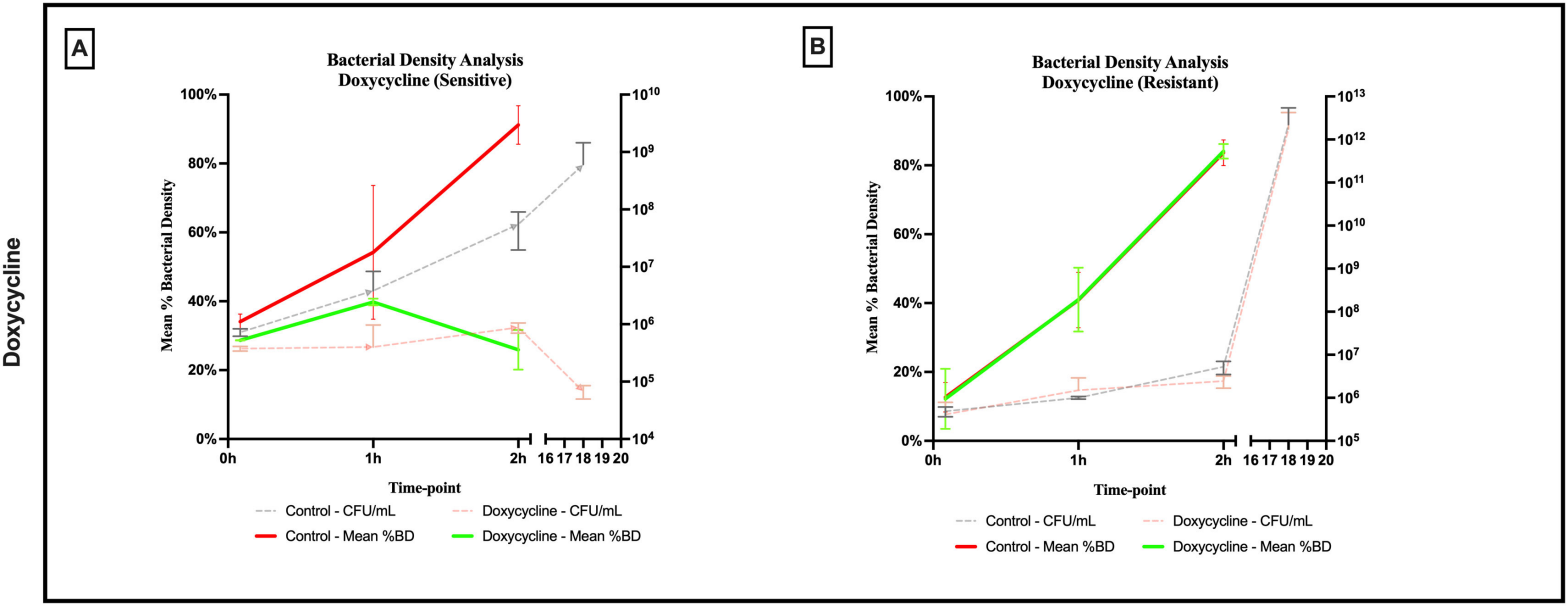
Percent bacterial density means analysis of bacteriostatic effect of doxycycline on **(A)** sensitive and **(B)** resistant strains of *E.coli* using scanning electron microscopy compared to culture colony counts (CFU). Error bars represent the standard deviation (SD).

**Figure 8:**
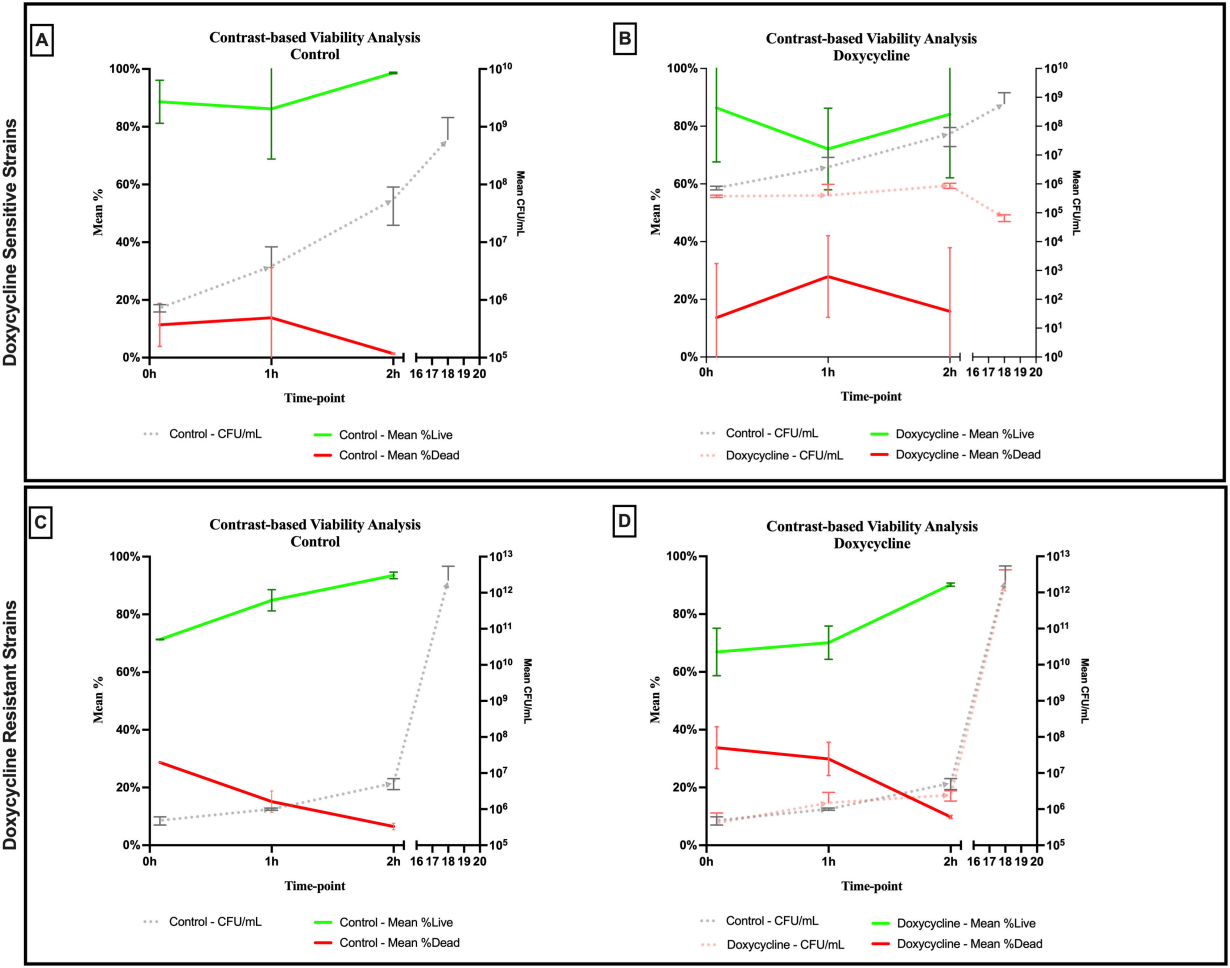
Contrast-based viability analysis of bacteriostatic effect of doxycycline on **(A, B)** sensitive and **(C, D)** resistant strains of *E.coli* using scanning electron microscopy compared to culture colony counts (CFU). Error bars represent the standard deviation (SD).

**Figure 9:**
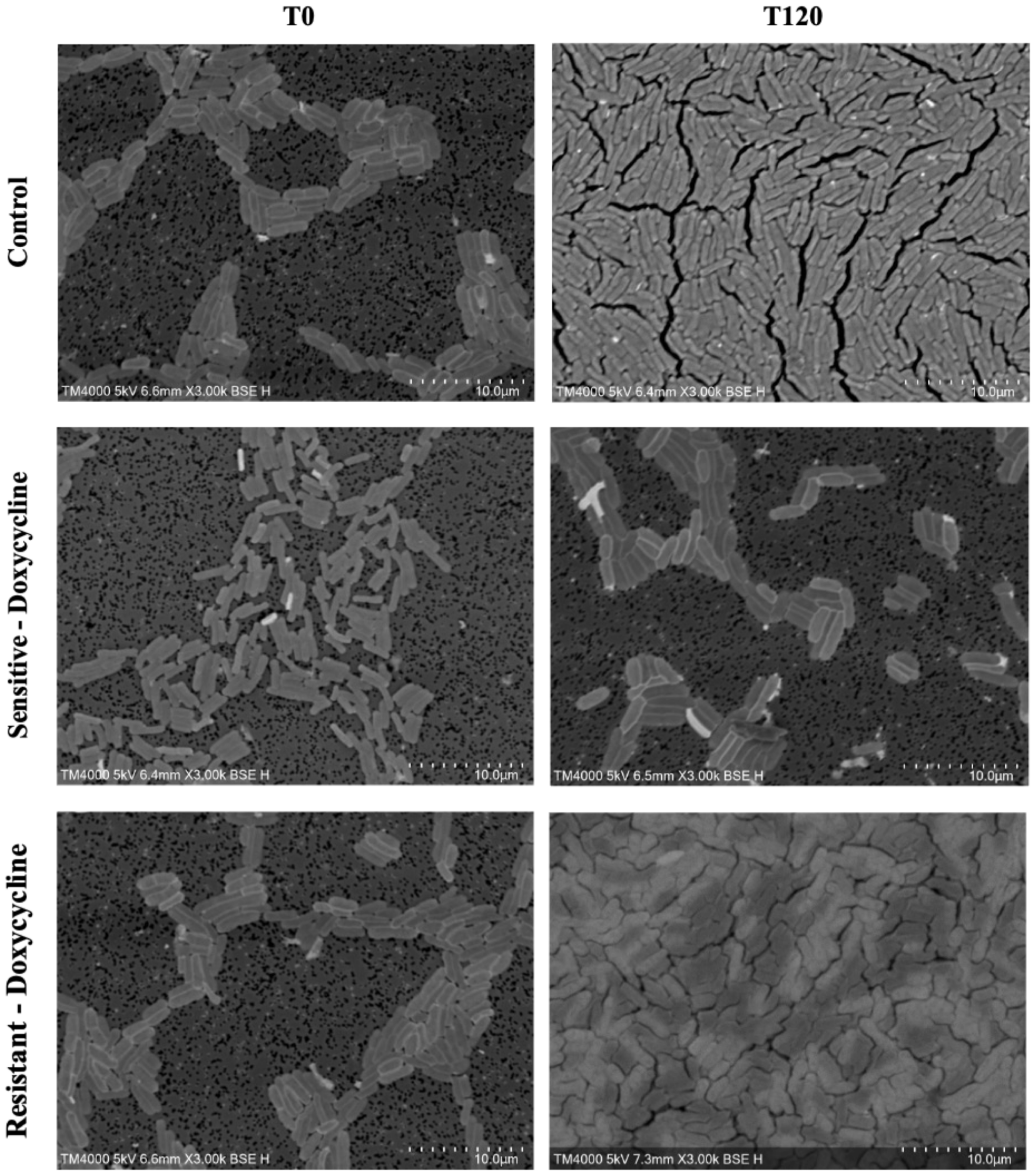
Morphologic/ultrastructural changes demonstrating the bacteriostatic effect of doxycycline on sensitive and resistant *E.coli* strains using scanning electron microscopy.

An evident stability was observed across all axes of our analyses for the Doxycycline sensitive strains of *E. coli*. An evident arrest in bacterial growth was observed starting 60 minutes following exposure to the antibiotic (**Figure 3**). Bacterial density analysis revealed a stagnation in mean %BD two hours following Doxycycline exposure (28.82% ± 0.28 to 23.24% ± 9.5) as compared to the increase in %BD in the control (34.08% ± 2.19 to 91.19% ± 5.58) (**Figure 7A**). Contrast-based viability analysis also revealed a slight variation in mean %Dead bacteria (22.96% ± 5.73 to 14.03% ± 19.49) in the Doxycycline exposed bacteria, with a similar change in the mean %Dead bacteria in the control (18.48% ± 0.59 to 4% ± 6.79) (**Figures 8A, B**). Also, following exposure to Doxycycline, we observed an absence of morphological changes at all time-points. (**Figure 9**) A parallel trend was observed in the culture colony counting method, revealing a mere 57% growth reduction at two hours, and an 82% growth reduction at 18 hours in the Doxycycline exposed bacteria, demonstrating a persistence of culturable bacteria at 18 hours despite antibiotic exposure, in contrast to the significant >3-log10 growth in the control. (**Figures 7**, **8**) Applying the SEM Predictive Score based on the combined results of the respective criteria provides a score of 0 (Bacteriostatic Effect) for all tested sensitive strains.

For the Doxycycline resistant *E. coli* strains, we observed an equivalent trend between the Doxycycline exposed bacteria and the controls. The resistant bacteria preserved their original morphology following exposure to Doxycycline. (**Figure 9**) Similarly, the culture colony count at 120 minutes showed a matching growth between the control and the Doxycycline exposed bacteria. This parallel trend was also observed in both %BD analysis (**Figure 7B**) and the contrast-based viability analysis. (**Figures 8C, D**) Calculating the SEM Predictive Score based on the combined results of the respective criteria provides a score of +1 (Resistant strain) for all tested resistant strains.

## 4 Discussion

In our study, we demonstrated the feasibility of using novel tabletop scanning electron microscopy to perform an early prediction of the bactericidal and bacteriostatic effects of Imipenem and Doxycycline, respectively, with *E. coli*. We further constructed a SEM Predictive Score (**Figure 2**) based on three criteria which are easily derived following our optimized automated image analysis methodology. This score allowed us to accurately identify the effect exerted by each antibiotic by relying on objective measures, including bacterial density, bacterial viability status, and morphologic/ultrastructural changes. These individual criteria are independently informative; however, we found that combining all three enhances our ability to accurately identify the antibiotic’s effect.

For our prediction of the bacteriostatic effect of Doxycycline, we demonstrated the utility of SEM in identifying an early onset arrest in the growth of bacteria, as visible at 60 minutes following incubation with Doxycycline. This was confirmed with the observed stable bacterial density on SEM micrographs, and stable CFU by culture. The persistence of growth of bacteria at 18 hours of incubation, with a growth reduction of <99.9%, validates our prediction in that our early observation of growth arrest, minimal fluctuation in bacterial viability, and preserved morphology are indeed predictive factors for the bacteriostatic effect.

As for our prediction of the bactericidal effect of Imipenem, we established a holistic representation of bacterial killing by the bactericidal agent, revealing major morphological and ultrastructural disruption of bacteria as early as 60 minutes following exposure to Imipenem. These changes were coupled to a striking increase in bacterial killing detected by our contrast-based viability analysis. Bacterial density analysis revealed minimal variation, attributable to the major impact of the morphological changes, in that despite the persistence of bacterial density, the majority of the observed bacteria were deformed and dead. This highlights the importance of combining all three criteria to define the bactericidal effect. This bactericidal effect was confirmed by the absence of growth using traditional culture colony counts at 18 hours, in line with the classic definition requiring >99.99% killing of bacteria (Pankey and Sabath, 2004). Therefore, our SEM predictive score has allowed the demonstration of the bactericidal effect of Imipenem through severe morphologic/ultrastructural changes, non-significant variation in bacterial growth density, and a flagrant rise in dead bacteria.

This novel tabletop technology brings forward a high-throughput approach using a compact instrument which will accelerate real-time monitoring of bacterial-antibiotic interactions, following simple and quick sample preparation, improving the definition of antibiotic effect through tangible and objective variables that are reachable at earlier time points; with minimized technical expertise and turnaround time, while uncovering a deeper dimension by revealing major ultrastructural modifications exclusive for each of these effects. This reduced time to result remains to be the holy grail factor for all microbiology methods. These faster methods provide a more nuanced understanding of antibiotic activity, capturing early events like membrane disruption or metabolic inhibition. In contrast to the routine MIC or MBC determination tests which reflect the bacteriostatic or bactericidal effect (Reller et al., 2009), we are able to identify early antibiotic effect on bacteria and provide an in-depth description of the bacterial interactions with the antibiotics in under 2 hours of incubation. We are also able to detect the level of killing, which correlated with the level of killing measured at 18 hours. There are several tests which can be performed to determine the antibiotic effect exerted on bacteria (Reller et al., 2009; Baranova et al., 2023), either using patient sera, or other methods. Apart from their uncertain clinical utility, most of these complex methods remain confined to research-use and are technically demanding or too costly for implementation in routine clinical settings (Peterson and Shanholtzer, 1992). To enhance our understanding of antibiotic effects, further research applying our method to a broader range of bacteria-antibiotic combinations is essential. This will facilitate a robust comparison with traditional methods, such as MIC and MBC, and help differentiate between antibiotics based on their specific mechanisms of action.

Furthermore, we must recognize the historical controversy over the utility of classifying antibiotics as bacteriostatic or bactericidal in the clinical setting (Pankey and Sabath, 2004; Nemeth et al., 2015), especially in relation to their impact on clinical outcomes. Multiple studies have attempted to demonstrate that this classification might append power to certain antibiotics, given their ability to rapidly kill bacteria in particular clinical situations such as endocarditis and sepsis (Pankey and Sabath, 2004), however, other studies contest these ideas and discuss the complexity involved in fighting an infection based on host, pathogen, and drug characteristics. A deeper understanding of immune responses coupled to antibiotic therapies is essential for determining the utility of such classifications (Stokes et al., 2019), but is beyond the scope of our work. Our focus is to provide a proof-of-concept for a practical SEM-based evaluation of bacteria-antibiotic interaction, which would eventually be useful in developing more complex assays using SEM which can help guide clinical decisions in a rapid and robust manner (Datar et al., 2021; Song and Lei, 2021). For example, we showed how the real-time observation allowed a rapid identification of resistant bacteria, which would greatly enhance antimicrobial stewardship efforts related to rapid de-escalation in therapy. Furthermore, on a research level, such detection of antibiotic effects can accelerate the determination of mechanisms of actions of antibiotics or other antimicrobial solutions under development (Song and Lei, 2021).

## 5 Conclusion

In conclusion, SEM-based prediction of the bactericidal and bacteriostatic effects of antibiotics on bacteria is a promising approach that is rapid, simple, and operator-independent. Further research exploring the mechanisms of action of more antibiotics on other bacterial species using SEM is essential. Testing a wider range of antibiotics and bacterial species will not only validate the robustness of the existing score but also holds the potential to uncover new criteria that could strengthen the performance of the score. This score-based approach is a strong candidate for machine learning applications, and will accelerate the development of artificial intelligence-assisted tools that will further expedite access to results.

## Data Availability

The original contributions presented in the study are included in the article/**Supplementary Material**. Further inquiries can be directed to the corresponding author.
