# Supplementary material for: Early prediction of the bactericidal and bacteriostatic effect of imipenem and doxycycline using tabletop scanning electron microscopy
Source: Front Cell Infect Microbiol. 2024 Aug 29;14:1431141. doi: 10.3389/fcimb.2024.1431141 (PMC11390654; doi:10.3389/fcimb.2024.1431141)
Supplement: Supplementary file 1 [file Table1.docx]

Supplementary Material

**
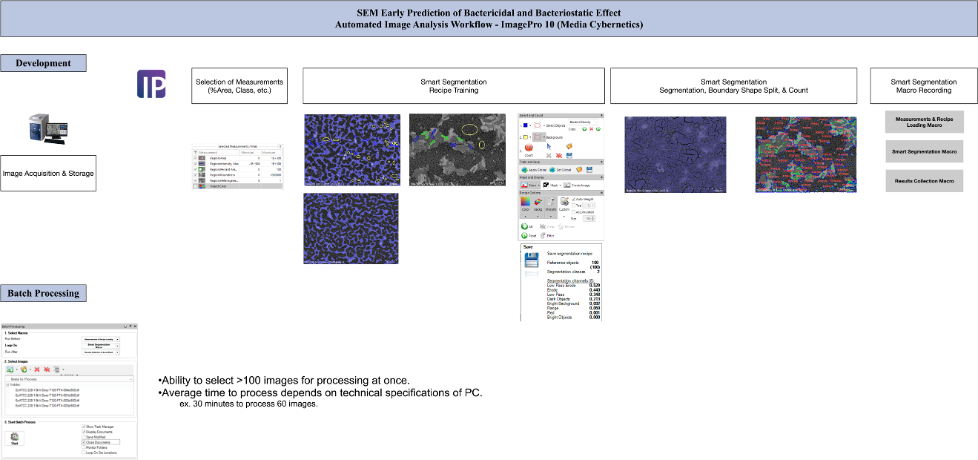
**

**Supplementary Data 1.** Automated Image Analysis Workflow using ImagePro 10 Developed for Early Prediction of Bactericidal and Bacteriostatic Effect using Scanning Electron Microscopy.
